# Understanding the pathophysiology of acute critical illness: translational lessons from zebrafish models

**DOI:** 10.1186/s40635-024-00595-x

**Published:** 2024-01-31

**Authors:** Kensuke Fujii, Kazuma Yamakawa, Yuriko Takeda, Natsuko Okuda, Akira Takasu, Fumihito Ono

**Affiliations:** 1 Department of Emergency and Critical Care Medicine, Osaka Medical and Pharmaceutical University, 2-7 Daigakumachi, Takatsuki, Osaka 569-8686 Japan; 2 Department of Physiology, Osaka Medical and Pharmaceutical University, 2-7 Daigakumachi, Takatsuki, Osaka 569-8686 Japan

**Keywords:** Zebrafish, Sepsis, Organ failure, Infection, Trauma, Regeneration

## Abstract

The models used to investigate the pathophysiological mechanisms of acute critical illness are not limited to mammalian species. The zebrafish (*Danio rerio*) is a popular model organism for studying diseases due to its transparency and rapid development. The genes and signaling pathways involved in acute critical illness appear highly conserved among zebrafish and humans. Forward genetics such as random mutagenesis by a chemical mutagen or reverse genetics methods represented by CRISPR/Cas9 allowed researchers to reveal multiple novel aspects of pathological processes in areas including infection, immunity, and regeneration. As a model of sepsis, transgenic zebrafish allowed the visualization of lipopolysaccharide (LPS)-induced vascular leakage in vivo and the demonstration of changes in the expression of cellular junction proteins. Other transgenic zebrafish visualizing the extravascular migration of neutrophils and macrophages have demonstrated a decrease in neutrophil numbers and an increased expression of an inflammatory gene, which replicates a phenomenon observed in humans in clinically encountered sepsis. The regenerative potential and the visibility of zebrafish organs also enabled clarification of important mechanisms in wound healing, angiogenesis, and neurogenesis. After spinal cord injury (SCI), a marker gene expressed in glial bridging was discovered. Furthermore, localized epithelial-to-mesenchymal transition (EMT) and molecular mechanisms leading to spinal cord repair were revealed. These translational studies using zebrafish show the potential of the model system for the treatment of acute critical illnesses such as sepsis, organ failure, and trauma.

## Introduction

Animal models are essential tools for research and provide a better understanding of the pathophysiology of acute critical illness. Since the 1930s, a small teleost zebrafish (*Danio rerio*) was developed and is now established as a model for development and regeneration studies [1]. Furthermore, it now became widely recognized as a model of human diseases, in areas including inflammation, infection, and drug discovery/screening among others [2].

**Fig. 1:**
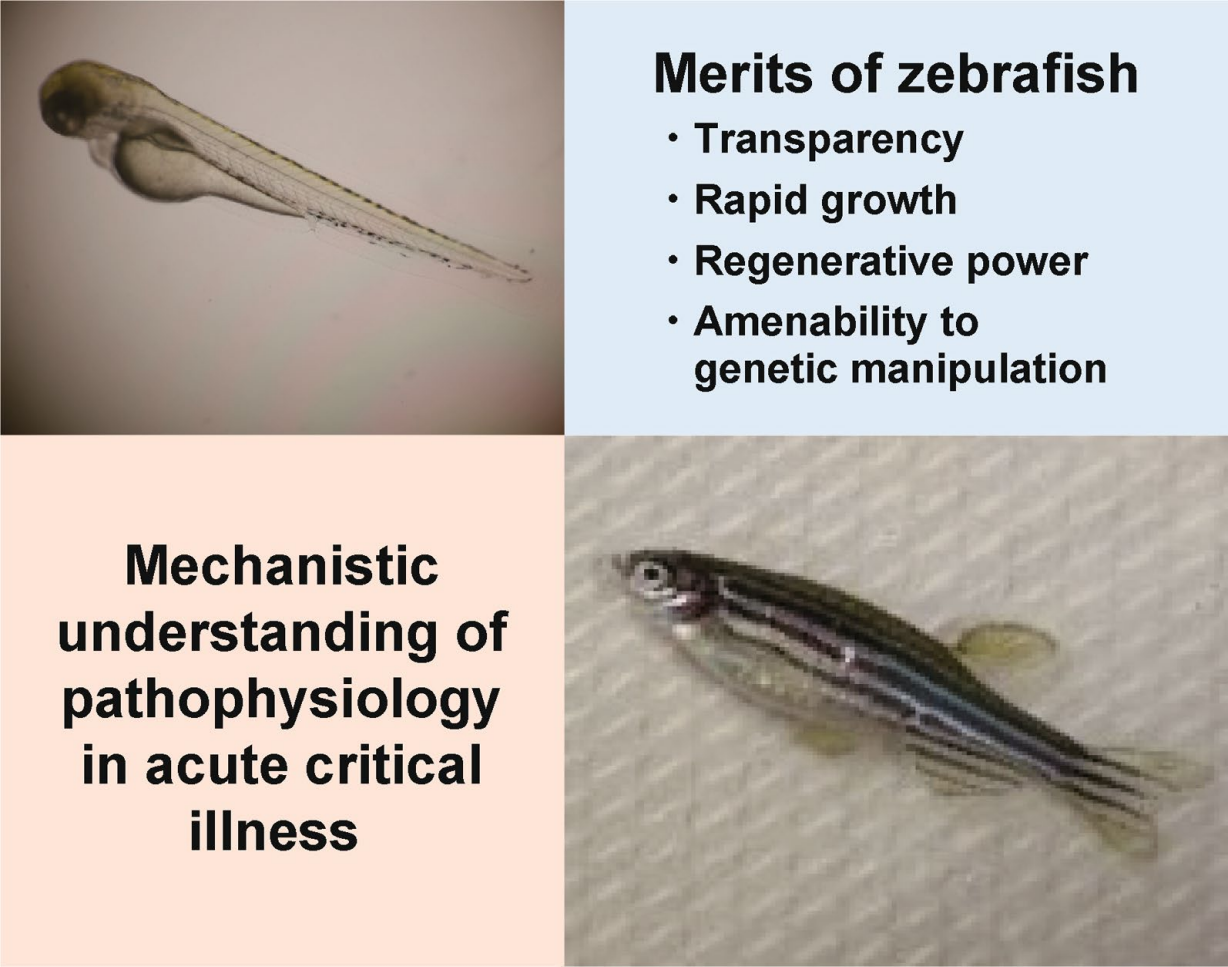
Novel pathophysiological mechanisms of acute critical illness newly revealed using zebrafish

Zebrafish have advantages over mammalian models in several aspects. Combined with genome-editing techniques, their characteristics enable the investigation of human diseases at a level unattainable in other model systems. There are four features of zebrafish that make them valuable for studying fundamental principles underlying acute critical illness. First, zebrafish share extensive genomic homology with humans. Among 3176 human genes associated with diseases, more than 80% are conserved in zebrafish [3]. Second, they are transparent and amenable to live imaging in vivo. Third, tools for genetic manipulation, including CRISPR/Cas9 and GAL4/UAS, have been established. Fourth, the organs in zebrafish display higher regenerative potential than those in mammals. These features allow researchers to visualize biological processes in vivo, which is difficult or impossible in other model systems (Fig. 1).

Zebrafish research excels in revealing conserved biological pathways and mechanisms, enhancing our understanding of human biology and diseases. Insights by clinical scientists contribute to uncover underlying biological basis of pathological processes in mechanistic detail [2]. In this review, we summarize the translational research on zebrafish, focusing on sepsis, organ failure, and trauma, and discuss technologies that are applicable in this model organism (Table 1).

**Table 1:** Summary of studies and findings discussed in the text

No.	Acute critical illness	Topics	Zebrafish models	Major findings	References
1	Sepsis	Pathogen-associated molecular patterns (PAMPs)	LPS-induced zebrafish endotoxemia	# Extravascular migration of neutrophils and macrophages # Change of expression in the tight junction protein Cldn5a/b, scaffold junction protein ZO-1 and a late mediator of inflammation HMGB1	[10]
2		Noncanonical inflammasome process, inflammatory caspase	Knocking out *caspy2* in zebrafish fibroblast cell Knockdown of *caspy2*	# Pyroptosis and caspy2 oligomerization binding directly to LPS # Noncanonical pathway determining mortality	[14]
4	Trauma	Pro-inflammatory cytokine in the healing process	*cloche* mutant	# Expression of *ilb2* in response to tissue amputation and in migrating cells # Function for *ilb2* and *irf8* in regulating regenerative cells apoptosis	[22]
5		Angiogenesis in wound healing	Transgenic expressing EGFR in endothelial cells	# Function of intraluminal pressure (IP) in angiogenesis # Role of IP sensor, TOCA family of F-BAR proteins	[23]
6		Cardiomyogenesis in injured heart	Myocardium-specific *klf1* knockdown model	# Role of Klf1 in proliferation of cardiomyocytes	[25]
7		Neurogenesis in injured brain	Stab injury model of optic tectum	# Radial glia (RG) cell regulating Wnt signaling	[27]
8		Spreading depolarization (SD) in brain	Recording SD model in tectum	# Role of NMDA receptors in SD	[28]
9		Neurogenesis in spinal cord injury	Disrupting/overexpressing connective tissue growth factor a (*ctgfa*) Knockouts of epithelial-to-mesenchymal transition (EMT)-driving genes Transgenic model with modified *hdac1* expression in progenitor cells	# *ctgfa* accelerating glial bridging and functional regeneration # Promotion of spinal cord repair through localized EMT # Promotion of regenerative neurogenesis through *hdac1* in spinal progenitors	[29–31]

## A model for sepsis

Sepsis, which is defined as life-threatening organ dysfunction due to a dysregulated host response to infection, requires urgent recognition due to its high lethality. It's the primary cause of death in human infections, and is distinguished from simple infections by an aberrant host response and organ dysfunction. Influenced by the combination of pathogen and host factors which include genetics, age, and comorbidities, sepsis can present with occult organ dysfunction, making it crucial to consider in any infection case. Additionally, unexplained organ dysfunction may suggest an underlying infection. The sepsis phenotype is affected by existing illnesses and treatments, and certain infections may cause localized organ dysfunction without triggering a systemic dysregulated host response [4].

Dysregulated host response caused by infectious stimuli activate innate immunity and cytokine release. In addition to infection, several noninfectious processes, including severe trauma, burns, and ischemic reperfusion injury, can induce a clinical condition analogous to sepsis [5]. The understanding of sepsis pathophysiology has progressed over the years, resulting in the establishment of concepts, such as pathogen-associated molecular patterns (PAMPs), damage-associated molecular patterns (DAMPs), and pattern recognition receptors (PRRs), revealing molecules involved in the process [6, 7]. Intracellular signaling is activated by DAMPs and PRRs, which work as ligands and receptors, leading to the production of cytokines, including tumor necrosis factor (TNF) and interleukins (ILs). Counterparts of mammalian PRRs, such as Toll-like receptors (TLRs) and nucleotide-binding oligomerization domain-like receptors (NLRs), also play important roles in the zebrafish host defense [8]. The similarity between zebrafish and humans led researchers to use zebrafish for studying bacterial, viral, and fungal pathogens that infect humans. Additionally, Gomes et al. pointed out the potential of integrating RNA sequencing and other 'omic' techniques in zebrafish studies, which can provide deeper insights into human infections [9].

**Fig. 2:**
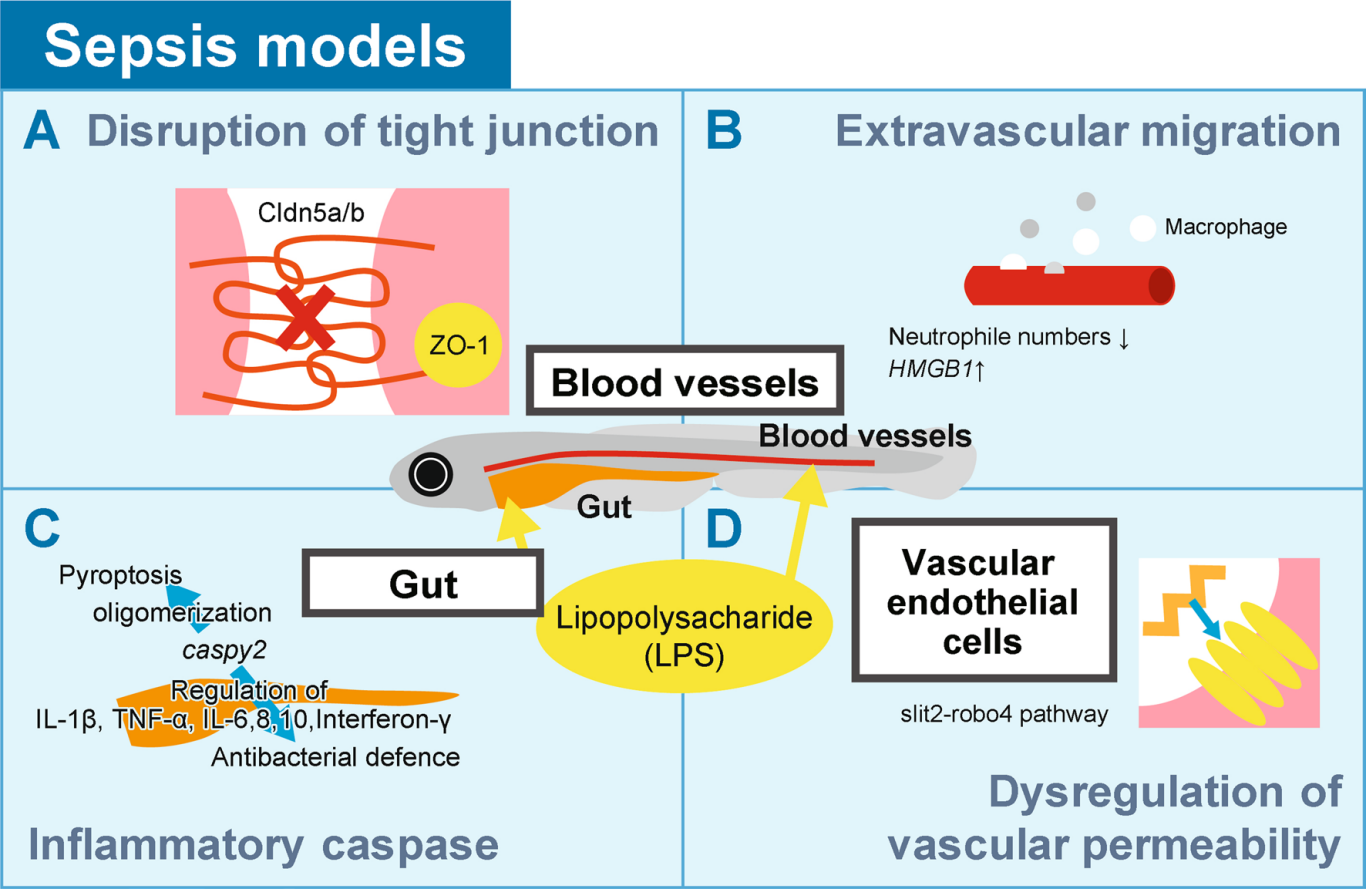
Sepsis models. **A**–**D** Sepsis and organ failure induced by lipopolysaccharide (LPS). The inflammatory caspase pathway in fibroblast cells and in the developing gut was revealed (**A**). Transgenic zebrafish lines displayed vascular leakage and changes in the expression of cellular junction proteins (**B**). In vivo, the *slit2-robo4* pathway regulated vascular permeability (**C**). The extravascular migration of neutrophils and macrophages was visualized using a transgenic zebrafish model (**D**)

Lipopolysaccharide (LPS) is a major constituent of the outer membrane of Gram-negative bacteria and is often used as a PAMP of exogenous origin in zebrafish as well as in murine sepsis models. Philip et al. used an LPS-induced zebrafish endotoxemia model to visualize subsequent phenomena in vivo [10] (Fig. 2A). They visualized LPS-induced vascular leakage with transgenic zebrafish lines and showed changes in the expression of cellular junction proteins. Embryos immersed in LPS showed a decrease in the expression of the tight junction protein *Claudin5a*/b (*Cldn5a/b)* and the associated scaffold junction protein *ZO-1*, which was consistent with the mouse sepsis model. *Cldn5* belongs to the claudin family, which are key components of tight junction strands on cell membranes. Tight junctions act as physical barriers and control the movement of solutes and water through the space between cells, particularly in epithelial and endothelial tissues. This gene's function is crucial in maintaining the selective permeability of these cell barriers, which is critical in various physiological and pathological conditions.

In addition, authors visualized the extravascular migration of neutrophils and macrophages using transgenic zebrafish lines with genetically encoded fluorescence-marked cells (Fig. 2B). The transgenic model exposed to LPS showed a decrease in whole-body neutrophil numbers and an increase in the expression of a late mediator of inflammation *HMGB1,* which plays a crucial role in regulating transcription and organizing DNA involved in inflammation processes. This finding is consistent with the immunosuppressive state at the late stage of sepsis in humans. Further in vivo analyses of cellular processes using optical techniques utilizing genetically encoded fluorescence-marked cells will provide new insights into sepsis.

In the zebrafish sepsis model, key pathophysiological features such as cytokine storms, tachycardia and endothelial leakage were also observed, similar to human sepsis [11]. A recent model proposes that the noncanonical inflammasome process involving inflammatory caspases is critical for septic shock in mammals [12, 13]. A zebrafish inflammasome study of *caspy2,* which has the highest homology to human *caspase-4/5*, is therefore interesting in this regard. *Caspase-5*, whose expression is regulated by interferon-gamma and lipopolysaccharide, is a member of the cysteine-aspartic acid protease, and is pivotal in the activation leading to apoptosis. It also regulates cell growth and differentiation by cleaving Max, a key component of the Myc/Max/Mad transcription regulation network. Yang et al. reported the activation of pyroptosis, a form of programed cell death resulting from inflammation, via caspase-5-like activity in zebrafish fibroblast (ZF4) cells infected with hemolysin-overexpressing *Edwardsiella piscicida* or in response to immunostimulatory molecules including LPS [14] (Fig. 2C). The critical role of *caspy2* in pyroptosis was demonstrated by knocking out (KO) *caspy2* in ZF4 cells using CRISPR/Cas9 genome-editing technology. They found that *caspy2* binds directly to LPS via the N-terminal pyrin death domain, resulting in caspy2 oligomerization. Reverse transcription polymerase chain reaction (RT‒PCR) and whole-mount in situ hybridization (ISH) targeting *caspy2* mRNA at the development stage further revealed that *caspy2* is highly expressed in the developing gut, in agreement with its critical role for gut inflammation and antibacterial defenses. In the sepsis model treated with LPS, knockdown of *caspy2* by MO (morpholino oligonucleotide) resulted in a higher survival rate in comparison to the control group. Finally, the significant upregulation of pro-inflammatory factors including IL-1β, TNF-α, IL-6, IL-8, IL-10 and interferon-γ was observed in the control-MO group in comparison to the *caspy2*-MO group, revealing that the noncanonical pathway determined mortality in vivo.

Zebrafish studies also identified targets of sepsis. Products of roundabout (robo) family genes bind to Slit and function classically in midline repulsion when axons extend toward the contralateral side [15]. In comparison to the classical robo structure with five immunoglobulin (IgG) and three fibronectin (FN) domains, robo4 is smaller. Murine robo4 contains two IgG and two FN domains, while zebrafish robo4 has three IgG domains. Specific domains in the cytoplasmic regions, whose structures are well studied, are also conserved between mammals and zebrafish [16, 17].

Unlike other family members expressed in neural tissues, *robo4* is critical in the cell–cell adhesion of angiogenesis and the establishment of the endothelial barrier. Its essential role in the vascular system was first reported in zebrafish [17]. Slit2-robo4 was later shown to be important in inflammation. During inflammation, it is downregulated in vascular endothelial cells by LPS, which leads to dysregulated vascular permeability [18] (Fig. 2D). Notably, human *robo4* can functionally compensate for the loss of *robo4* in zebrafish, despite the motif difference mentioned above. Based on these discoveries, it is now generally accepted that *robo4* in vascular endothelial cells functions in inflammation as well as in angiogenesis [19]. These studies highlight the power of the zebrafish model in revealing septic processes and involved molecules in vivo.

## Models of trauma and regeneration

In human patients, acute critical illness does not necessarily respond to treatment. In such unresponsive cases, organ failure can ensue. Endogenous diseases and trauma can also contribute to organ failure. Therefore, tissue/organ regeneration is an attractive topic in acute critical medicine research.

Zebrafish have the ability to regenerate complex structures such as fins, heart, brain, retina, and other organs [20], which provides ample opportunities to study regeneration mechanisms. It allowed researchers to explore genes and signaling pathways involved in regeneration in a vertebrate model. We highlight some of the recent studies on the process leading to wound healing and organ regeneration after acute critical illness.

**Fig. 3:**
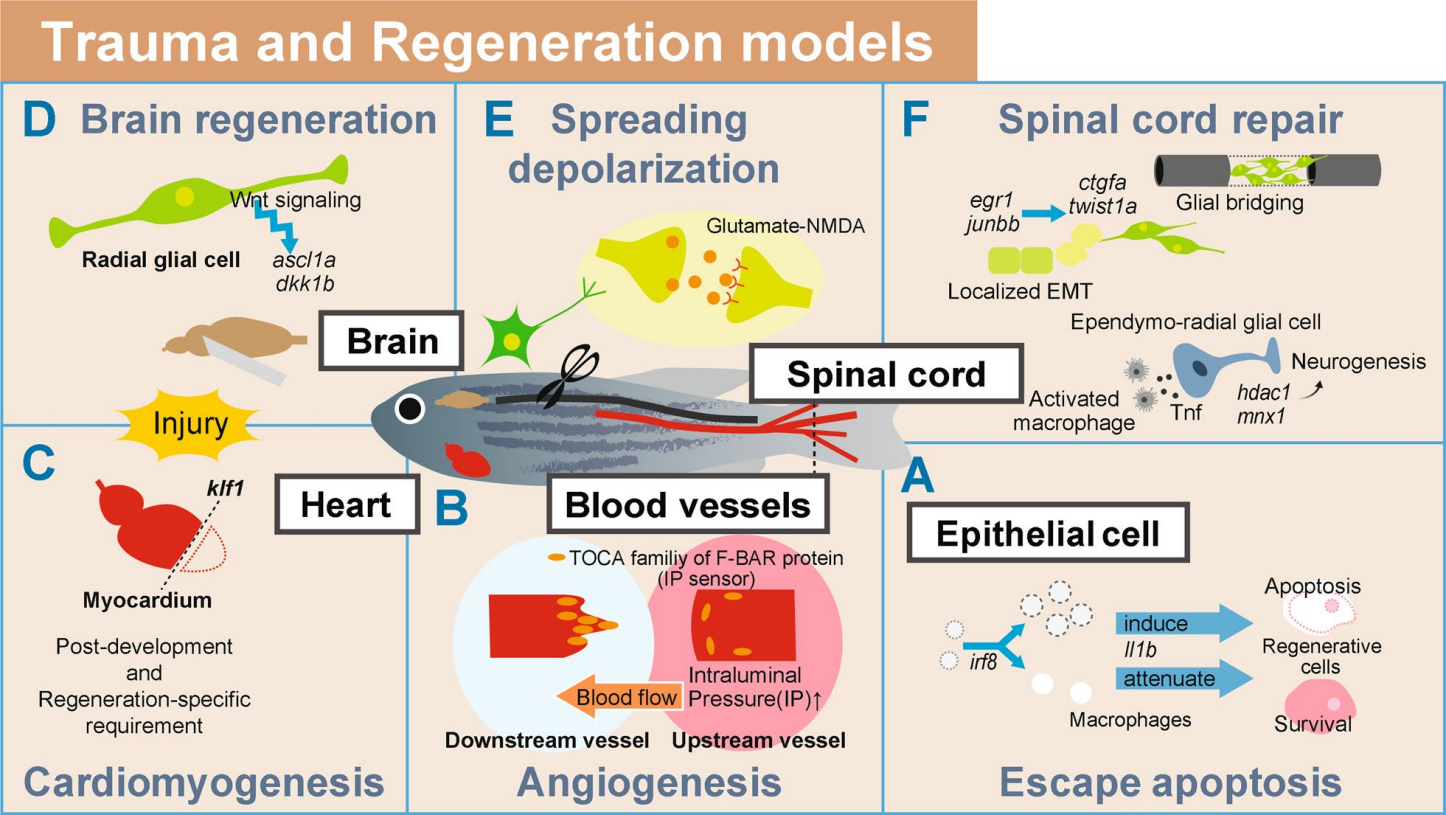
Trauma and regeneration models. **A**–**C** Regeneration after injury. The *il1b* expression in epithelial cells regulated the apoptosis of regenerative cells (**A**). In angiogenesis after vessel injury, intraluminal pressure (IP) sensors in epithelial cells regulate elongation (**B**). In the injured heart, the expression of *kl1a* was essential for cardiomyogenesis (**C**). **D**–**F** Damage to the central nervous system. Wnt signaling in radial glial cells regulated brain regeneration (**D**). In adult zebrafish brains, glutamate and its receptor were important for the propagation of spreading depolarization (**E**). In spinal cord injury (SCI), the upregulation of *ctgfa* in bridging glia was identified. Localized epithelial-to-mesenchymal transition (EMT) was further revealed to be the key factor in spinal cord regeneration. Tnf is a direct signaling axis from activated macrophages to spinal progenitor cells and promotes regenerative neurogenesis (**F**)

Zebrafish mutant *cloche* (*clo*), identified by forward genetics screening to have a defect in the transcription factor *npas4l* required for primitive hemopoiesis, is unable to regenerate its fin fold due to apoptosis of the regenerative cells [21] (Fig. 3A). IL-1β (*il1b*) was expressed, as observed by in situ hybridization (ISH), in more advanced stages of the healing process in the *clo* mutant, indicating a prolonged inflammatory reaction [22]. A pro-inflammatory cytokine IL-1β is produced by myeloid cells and plays a crucial role in the inflammatory response. After the injection of LPS, in contrast, fewer *il1b*-positive cells were identified in the *clo* mutant, myeloid defective zebrafish mutants. This unexpected observation indicated that tissue injury and bacterial infection induce the expression of *il1b* through distinct mechanisms.

When the expression of *il1b* was monitored using a transgenic line in which GFP was driven by the *il1b* promoter, the expression of *il1b* was induced in epithelial cells in response to tissue amputation and in migrating cells, which were likely myeloid cells. These data suggest that *il1b* plays pro-regenerative roles.

Paradoxically, in *clo* mutants in which *irf8*, a factor required for macrophage differentiation, was inhibited by morpholino (MO), *il1b* in epithelial cells caused the persistence of strong inflammation around the injury site and the apoptosis of regenerative cells. The interpretation was that regenerative cells escape apoptosis when macrophages attenuate *il1b*, leading to the rapid termination of inflammation and regeneration. These “double-edged” functions of *il1b* underscore the complex mechanism regulating regeneration.

Some studies on wound healing visualized angiogenesis in live zebrafish. Yuge et al. used adult transgenic zebrafish expressing EGFP in endothelial cells (ECs) to observe angiogenesis during cutaneous wound healing in adults and embryos [23] (Fig. 3B). This revealed that blood vessel regeneration is strikingly different between upstream and downstream vessels of the blood flow relative to the severed site. The regeneration occurred in the downstream vessels but not in the upstream vessels. Furthermore, experiments visualizing particles of different sizes in the injured area revealed that blood flow-driven intraluminal pressure (IP) applied in upstream blood vessels inhibited elongation. This surprising finding that IP regulates angiogenesis is in contrast to the conventionally held idea that hypoxia is the main driver.

When IP stretches ECs upstream of injured vessels, the TOCA (Transducer of Cdc42 dependent actin assembly) family of F-BAR (Fes/Cdc42 interacting protein 4 (CIP4) homology-BAR) proteins acts as sensors for IP. The TOCA family proteins remodel lipid bilayers through their F-BAR domains, while regulating actin dynamics via the SH3 domains. In upstream vessels, F-BAR proteins suppress the formation of actin-based membrane protrusions at the leading edge, inhibiting the establishment of front-rear polarity, the migration of ECs and the elongation of vessel sprouts. These results stress the close association of wound healing and regeneration.

## Trauma and regeneration in hearts

When hearts are injured, adult zebrafish can regenerate lost myocardium by altering the contractile structure of mature cardiomyocytes, which proliferate after undergoing temporary cardiomyocyte dedifferentiation. Krüppel-like factor 1 (klf1), originally known for its role in erythropoiesis [24], encodes a hematopoietic-specific transcription factor that strongly induces expression of erythroid genes. It turned out to be essential also in myocardial regeneration of adult zebrafish [25] (Fig. 3C). Klf1 colocalized with the cytosolic muscle marker troponin C and was expressed in the myocardium during regeneration. The expression was attenuated at the end of cellular regeneration. When myocardium-specific *klf1* knockdown was performed with a cardiac Cre-driver line (*klf1*-mKD), regeneration was compromised in these fish due to reduced dedifferentiation and proliferative cardiomyocytes in injured *klf1*-mKD hearts. Uninjured hearts in fish overexpressing *klf1* showed increased depolarization and proliferation of cardiomyocytes, a fivefold increase in cardiomyocytes, and myocardial hypertrophy. The cardiomyogenic potency of klf1 was thus proven, which provides highly interesting translational potential for organ regeneration after acute critical illness. The human heart, which has poor regenerative potential in comparison to zebrafish, may also benefit from further studies on klf1.

## Trauma and regeneration in the central nervous system

Among acute critical illnesses, damage to the central nervous system caused by trauma greatly impairs a patient’s quality of life [26]. Zebrafish have the capability to generate new neurons in the adult brain, and studying this process has great potential in supporting the development of therapies for devastating trauma.

A stab injury model of the optic tectum in zebrafish revealed that one type of neural stem cell, the radial glia (RG) cell, functions in regeneration [27] (Fig. 3D). After stab injuries, the expression level of *ascl1a* and *dkk1* changed in RGs. Both genes are involved in the Wnt signaling cascade, the former playing a role in the neuronal commitment and differentiation and the latter implicated in embryonic development and bone formation. Wnt signaling inhibitor treatment suppressed the proliferation and differentiation of RGs after stab injury. Thus, the regeneration of optic tectum after trauma occurs through the upregulation of Wnt signaling in RGs, which generates new neurons. This model is therefore valuable in revealing the molecular mechanisms of brain regeneration.

The brain of adult zebrafish is useful for simulating other clinical contexts. In migraine, stroke and traumatic brain injury, molecules triggering cellular excitation, such as glutamate and potassium ions, are released extracellularly from damaged neurons. The excitement slowly propagates to surrounding cells, compromising the energy metabolism and the blood flow, which worsens the damage in the injured brain. While this concept, known as spreading depolarization (SD), is important in clinical medicine, the molecular and cellular mechanism remains largely unknown. Terai et al*.* applied electrophysiological recordings in immobilized adult zebrafish to examine the excitability of the optic tectum and succeeded in recording SD in the adult zebrafish tectum [28] (Fig. 3E). This experimental system confirmed in the zebrafish model that glutamate (an excitatory neurotransmitter) and its receptor, *N*-methyl-d-aspartate (NMDA) glutamate receptor, are important for the propagation of spreading depolarization in tectal SD. This model may help establish a clinically applicable diagnostic technique for SD and its treatment.

Several studies using zebrafish aimed to develop regenerative therapies for traumatic spinal cord injury. Instead of generating scarring after spinal cord injury, the spinal cord of adult zebrafish regenerates in 6–8 weeks due to the pro-regenerative responses of glial cells, which form a bridge across the injured area and provide a scaffold for axonal growth.

Mokalled et al*.* performed transcriptome screening and identified a gene, connective tissue growth factor a (*ctgfa*), that is upregulated in bridging glia after spinal cord injury [29] (Fig. 3F). Disrupting this gene inhibited spinal cord repair, and conversely, overexpressing it accelerated glial bridging and functional regeneration. To pursue downstream events regulated by *ctgfa*, RNA-seq of *ctgfa*-positive cells was performed after spinal cord injury. The results revealed the upregulation of genes implicated in the epithelial-to-mesenchymal transition (EMT) [30]. They further established stable mutant lines in which these EMT-driving genes (*egr1, junbb*), both transcription factors, were knocked out with CRISPR‒Cas9. Remarkably, glial bridging and swimming duration were decreased in these mutants. In addition, when the expression of a gene promoting EMT, *twist1a*, a transcription factor known to play a role in the neural tube closure*,* was driven in transgenic (*hsp70*:Twist1a-2A-EGFP) zebrafish by heat shock, glial bridging and functional regeneration were promoted through EMT. These data showed that localized EMT was the key factor in regeneration after spinal cord injury. They further showed that this EMT-driving gene regulatory network distinguishes the regenerative zebrafish spinal cord from that of mammals. Glial bridging and the resultant functional spinal cord repair may be applicable to human therapy in the future.

Post-injury activated *tnfa*+ macrophages in zebrafish, similar to mammals, express high levels of cathepsin, marking them as reactive in the CNS regeneration (Fig. 3F). The *tnfa* gene produces a pro-inflammatory cytokine belonging to the tumor necrosis factor superfamily. This multifunctional cytokine plays a key role in regulating a broad range of biological processes, including cell proliferation, differentiation, apoptosis, lipid metabolism, and coagulation. *Tnfa* directly stimulates spinal neurogenesis, indicating that *tnfa*+ macrophages play a regenerative role. Tnf signaling boosts expression of *mnx1*, which is essential for motor neuron differentiation, and may reactivate neurogenesis. Additionally, expression of histone deacetylase 1 (*hdac1*) induced by tnfa could reprogram ependymo-radial glias (ERGs) for neurogenesis. Understanding *hdac1* and *mnx1* upregulation in (ERG) progenitors following immune activation sheds light on intracellular processes in spinal progenitor cells post-injury [31].

## Conclusion

Recent reports took advantage of the biological characteristics of zebrafish to study infection, immunity, angiogenesis, wound healing, and organ regeneration. The zebrafish, with its regenerative power and the applicability of various technologies, provides an attractive translational platform that may provide a path to the treatment of acute critical illness in humans.

## Data Availability

N/A.
